# Recent Initiatives in the Republic of Srpska to Enhance Appropriate Use of Antibiotics in Ambulatory Care; Their Influence and Implications

**DOI:** 10.3389/fphar.2018.00442

**Published:** 2018-05-29

**Authors:** Ljubica Bojanić, Vanda Marković-Peković, Ranko Škrbić, Nataša Stojaković, Mirjana Ðermanović, Janja Bojanić, Jurij Fürst, Amanj B. Kurdi, Brian Godman

**Affiliations:** ^1^Public Health Institute, Banja Luka, Bosnia and Herzegovina; ^2^Department of Pharmacy, Faculty of Medicine, University of Banja Luka, Banja Luka, Bosnia and Herzegovina; ^3^Ministry of Health and Social Welfare, Banja Luka, Bosnia and Herzegovina; ^4^Department of Social Pharmacy, Faculty of Medicine, University of Banja Luka, Banja Luka, Bosnia and Herzegovina; ^5^Department of Clinical Pharmacology, Faculty of Medicine, University of Banja Luka, Banja Luka, Bosnia and Herzegovina; ^6^Department of Epidemiology, Faculty of Medicine, University of Banja Luka, Banja Luka, Bosnia and Herzegovina; ^7^Health Insurance Institute of Slovenia, Ljubljana, Slovenia; ^8^Strathclyde Institute of Pharmacy and Biomedical Sciences, University of Strathclyde, Glasgow, United Kingdom; ^9^Department of pharmacology and toxicology, College of Pharmacy, Hawler Medical University, Erbil, Iraq; ^10^Liverpool Health Economics Centre, Liverpool University, Liverpool, United Kingdom; ^11^Division of Clinical Pharmacology, Karolinska Institute, Karolinska University Hospital Huddinge, Stockholm, Sweden; ^12^School of Pharmaceutical Sciences, Universiti Sains Malaysia, Kota Bharu, Malaysia

**Keywords:** antibiotic utilization, antibiotic resistance, cross national comparative study, initiatives, Republic of Srpska, quality indicators

## Abstract

**Introduction:** There are increasing concerns world-wide with growing rates of antibiotic resistance necessitating urgent action. There have been a number of initiatives in the Republic of Srpska in recent years to address this and improve rational antibiotic prescribing and dispensing despite limited resources to fund multiple initiatives.

**Objective:** Analyse antibiotic utilization patterns in the Republic of Srpska following these multiple initiatives as a basis for developing future programmes in the Republic if needed.

**Methods:** Observational retrospective study of total outpatient antibiotic utilization from 2010 to 2015, based on data obtained from the Public Health Institute, alongside documentation of ongoing initiatives to influence utilization. The quality of antibiotic utilization principally assessed according to ESAC, ECDC, and WHO quality indicators and DU 90% (the drug utilization 90%) profile as well as vs. neighboring countries.

**Results:** Following multiple initiatives, antibiotic utilization remained relatively stable in the Republic at 15.6 to 18.4 DIDs, with a decreasing trend in recent years, with rates comparable or lower than neighboring countries. Amoxicillin and the penicillins accounted for 29–40 and 50% of total utilization, respectively. Overall, limited utilization of co-amoxiclav (7–11%), cephalosporins, macrolides, and quinolones, as well as low use of third and fourth generation cephalosporins vs. first and second cephalosporins. However, increasing utilization of co-amoxiclav and azithromycin, as well as higher rates of quinolone utilization compared to some countries, was seen.

**Conclusions:** Multiple interventions in the Republic of Srpska in recent years have resulted in one of the lowest utilization of antibiotics when compared with similar countries, acting as an exemplar to others. However, there are some concerns with current utilization of co-amoxiclav and azithromycin which are being addressed. This will be the subject of future research activities.

## Introduction

The discovery of antibiotics is one of the greatest discoveries of modern medicine. However, since their discovery, it is acknowledged that bacteria have the ability to become resistant to antibiotics if they are not used rationally (Rezal et al., 2015; Godman et al., 2017). Excessive and non-rational use of antibiotics have resulted in increasing rates of antimicrobial resistance (AMR) worldwide, with ongoing initiatives to address this including addressing issues of governance (WHO, 2011, 2015; Collignon et al., 2015; Shallcross et al., 2015; Jinks et al., 2016). This is essential given the low number of new antibiotics in development; although there are activities to try and improve the situation (O'Neill, 2015; Tacconelli et al., 2018). Irrational use of antibiotics includes the prescribing and dispensing of antibiotics for upper respiratory tract infections (URTIs), which are the principal infections seen in ambulatory care and are predominantly viral in origin (Llor and Bjerrum, 2014; Dyar et al., 2016; Aabenhus et al., 2017). Inappropriate prescribing, as well as dispensing without a prescription, are enhanced by patient pressure, sub-optimal knowledge about antibiotics and viral infections among key stakeholder groups and fears among physicians and pharmacists that if they do not prescribe or dispense an antibiotic patients will go elsewhere (Jorgji et al., 2014; Yu et al., 2014; Hassali et al., 2015; Bai et al., 2016; Eslami et al., 2016; Kibuule et al., 2016; Chang et al., 2017).

The costs of AMR in Europe were estimated at €1.5 billion in 2007, but now rising to €9 billion per year or higher (Oxford and Kozlov, 2013; Gandra et al., 2014). The lack of new antibiotics alongside increasing AMR rates has resulted in increasing morbidity, mortality and costs to healthcare systems (Aminov, 2010; Gandra et al., 2014; O'Neill, 2015). As a result, AMR is now seen as one of the biggest public health challenges (ECDC, 2016), with deaths due to resistant strains envisaged to reach up to 444 million patients by 2050 if AMR is not addressed (Taylor et al., 2014; Fitchett and Atun, 2016). This is preventable with the spread of resistant strains directly correlated with increasing and irrational use of antibiotics (Costelloe et al., 2010; Bell et al., 2014; Llor and Bjerrum, 2014). Consequently, raising awareness and knowledge about AMR and rational antibiotic use among all key stakeholder groups should result in a reduction in AMR rates (Huttner et al., 2010; Md Rezal et al., 2015; Rezal et al., 2015; Godman et al., 2017; WHO, 2018). We are already seeing multiple activities across Europe including former Soviet Union Republics to tackle concerns with current antibiotic use and this will continue (Huttner et al., 2010; Fürst et al., 2015; Abilova et al., 2018; ECDC, 2018).

The Republic of Srpska is one of the two constituent entities of Bosnia and Herzegovina, with an estimated population of ~1.4 million. The Republic has implemented a range of laws and other activities over the years to regulate health care activities, which includes enhancing the appropriate use of medicines through initiatives including introducing good clinical practice guidelines (MoHSW, 1999, 2008, 2009, 2010; PABH, 2008; Petrusic and Jakovljevic, 2015). Activities have also been ongoing in other Balkan countries; however, there are concerns with the practice of evidence based medicine, the cost consciousness of physicians even after cost containment policies as well as the principles of resource allocation among physicians, although this is changing (Jakovljevic et al., 2016a,b). There are also concerns with the level of co-payments among patients in the Balkan countries especially with high cost medicines; although this has not prevented pharmaceutical markets growing among the Balkan countries as seen in Bulgaria, Croatia, the Republic of Srpska and Serbia in recent years, including medicines for cardiovascular and respiratory diseases, cancer and depression (Putrik et al., 2014; Jakovljevic et al., 2015; Petrusic and Jakovljevic, 2015; Jakovljevic and Souliotis, 2016; Kostić et al., 2017; Pejcic and Jakovljevic, 2017).

Policies and programmes to improve the health of patients with limited resources in the Republic of Srpska include improving the availability and affordability of medicines, improving antibiotic prescribing and dispensing, and raising awareness on excessive antibiotic utilization and resistance. A summary of these initiatives are contained in Boxes 1, 2. The various activities have increased prescribing efficiency as well as enhanced prescribing in accordance with standard treatment guidelines, positively impacting on for instance the rate of polypharmacy in practice (Markovic-Pekovic et al., 2012; Marković-Peković et al., 2016).

Box 1 **Legal framework for regulation of health care and rational medicine use in the Republic of Srpska.**

- Laws, rulebooks, programs, and policies for regulating the health care and rational use of medicines, including antibiotics, adopted by Ministry of Health and Social Welfare (MoHSW) of the Republic of Srpska (MoHSW, 1999, 2008, 2009, 2010; PABH, 2008).
- Antibiotics can only be dispensed on prescription by pharmacist. There are considerable fines if this is abused (MoHSW 2008; PABH, 2008).
- The Republic of Srpska Inspectorate is the responsible institution for supervising the implementation of this legislation.
- The MoHSW established the National Committee for AMR Control as a national expert body (2015) responsible for monitoring and controlling of AMR and proposing measures to improve rational antibiotic prescribing and utilization.
- On the proposal of the National Committee, the MoHSW adopted the national Program to reduce AMR resistance in the Republic of Srpska from 2016 to 2020, harmonized with the WHO Global Strategy for Containment of Antimicrobial Resistance (WHO, 2001) and the Council of Europe Recommendation's on prudent use of antimicrobial agents in human medicine (EC, 2002). The aim of this program is to establish and implement high-quality and effective health care for the population ensuring good control of AMR with an emphasis on rational antibiotic use. The stated objectives are to be reached through improving the intersectoral control on AMR and antibiotic use in healthcare as well as among veterinary institutions, seeking to reduce morbidity and mortality caused by multiresistant strains of bacteria, improving education among health workers and associates in the field of AMR, raising awareness on AMR and rational antibiotic use and, also, through participation of representatives from the Republic of Srpska in international networks and research in the field of AMR.

Box 2 **Activities to improve rational antibiotic use in the Republic of Srpska.**

**Activities to enhance rational prescribing**

- The set of Clinical Guidelines (CG) with diagnostic and therapeutic principles have been developed for Primary Health Care physicians. These include guidelines for upper and lower acute respiratory infections in children (acute otitis media, tonsillopharyngitis, community acquired pneumonia), urinary tract infections in children, as well as urinary tract infections in adults (2004).
- The list of antibiotics funded by the Health Insurance Fund of the Republic of Srpska (HIF) are in accordance with the CG recommendations.
- HIF adopted a positive list of medicines with reference prices comprised of List A and List B. List A is a basic list of medicines for which HIF covers 90 or 100% of the reference price of the medicine depending on the category of the insured person. List B is a supplementary list, which includes more expensive medicines, for which HIF covers 50% of the costs for all insured patients. For medicines not included in the positive list, the patient must pay 100% of the price. In addition, if the price of drug is higher than the reference price adopted by HIF, the patient has to pay the price difference.
- The antibiotics included in List A are: doxycycline, amoxicillin, phenoxymethylpenicillin, benzathine-phenoxymethylpenicillin, cefalexin, sulfamethoxazole with trimethoprim, and erythromycin. Norfloxacin is included in List B.

**Activities to enhance rational dispensing**

- There is permanent education of pharmacists and pharmacy technicians on the appropriate management of infectious diseases to address concerns with knowledge of infectious diseases, especially viral infectious diseases, which has been a concern in other countries (Eslami et al., 2016; Saleem et al., 2016; Belkina et al., 2017; Hoxha et al., 2018).
- The Pharmaceutical Association of the Republic of Srpska launched the ‘*The Guideline for counseling patients in the pharmacy*’ as a tool to assist pharmacy personnel to make decisions whether they can successfully treat patients with non-pharmacological measures and/or with OTC medicines, or whether to refer them to a family practitioner, in view of concerns with self-medication with antibiotics in the Republic (Marković-Peković and Grubisa, 2012; Damjanović et al., 2013; Marković-Peković et al., 2017).

**Activities of professional associations and key stakeholder groups**

- Regular professional meetings, conferences and symposia in order to upgrade the knowledge, habits and attitudes of health professionals (doctors and pharmacists).
- Publishing articles on AMR and rational antibiotic use in professional journals.
- Discussions concerning current AMR surveillance in the Republic of Srpska among key stakeholder groups and agreement of specific activities to improve AMR reporting and monitoring.
- The MoHSW and the Public Health Institute (PHI) marking European Antibiotic Awareness Day and World Antibiotic Awareness Week in November each year starting from 2013 with support of European Centre for Disease Prevention and Control (ECDC).

**Activities to enhance public awareness on AMR and rational antibiotic use**

- Campaigns are organized to raise awareness about AMR and rational antibiotic use among the general public, patient associations and health professionals.
- Promotional materials (including flyers, infographics, fact sheets, posters, and social media), recommended by ECDC, are provided to patients and health professionals.

Consequently, the aim of this study was to analyse total antibiotic utilization in the Republic of Srpska in recent years and to assess the influence of these various initiatives to improve antibiotic prescribing and dispensing in the Republic. Subsequently, compare the findings with those of other European and neighboring countries to review potential additional measures that could be introduced in the Republic of Srpska if needed to further improve the rational use of antibiotics. As a result, seek to reduce future AMR rates.

## Methods

This was a retrospective observational study on outpatient antibiotic utilization in the Republic of Srpska from 2010 to 2015, based on drug utilization data obtained from the Public Health Institute (PHI). Data collection was based on quantitative and structural medicinal reports that pharmacies in the Republic send to the PHI each year for collation (Petrusic and Jakovljevic, 2015). Consequently, includes total antibiotic utilization in the Republic and not just prescribed antibiotics.

Drug utilization analysis of the PHI data was undertaken using the ATC (Anatomical Therapeutic Chemical classification)/DDD (Defined Daily Dose) methodology (WHO, 2017), which is the internationally accepted methodology for measuring medicine utilization within and across populations (Godman et al., 2014; Malo et al., 2014; Versporten et al., 2014; Abilova et al., 2018). DDDs are defined as the amount of drug most commonly used in adults for the most common indication. It is a suitable measure to describe and compare drug utilization patterns between different geographical areas and health facilities. Data on outpatient antibiotic utilization are expressed in DDD/1,000 inhabitants/day (DIDs) for comparative purposes (Malo et al., 2014; Versporten et al., 2014; WHO, 2017). DU 90% (drug utilization 90%) was also used as indicator for assessing the quality of antibiotic prescribing, ranking antibiotics first by volume of DIDs and then by how many antibiotics were included in DU 90% (Markovic-Pekovic et al., 2009; Malo et al., 2014).

Antibiotic utilization and associated quality indicators were used to compare the Republic of Srpska's utilization patterns with that from other European countries, as well as former Soviet Union Republics, to place this in perspective (Adriaenssens et al., 2011a,b; Versporten et al., 2014; de Bie et al., 2016; WHO EUROPE, 2017). The quality of antibiotic prescribing was assessed using the European Surveillance of Antimicrobial Consumption (ESAC), European Centre for Disease Prevention and Control (ECDC), and World Health Organization (WHO) Europe quality indicators (Adriaenssens et al., 2011a,b; WHO EUROPE, 2017; ECDC, 2018). These included:

- Total utilization of antibiotics expressed as DIDs,
- Utilization of penicillins (J01C) as a % of total antibiotic use,
- Penicillins combination utilization such as amoxicillin with clavulanic acid (co-amoxiclav) as a percentage of total antibiotic use,
- Total utilization of cephalosporins expressed as DIDs,
- Percentage utilization of third- and fourth-generation of cephalosporins vs. all cephalosporins,
- Total utilization of J01F group (macrolides, lincosamides, and streptogramins) expressed as DIDs
- Utilization of short-acting macrolides, intermediate and long-acting macrolides (erythromycin, clarithromycin, and azithromycin) as a percentage of total antibiotic use,
- Utilization of quinolones (J01M) expressed in DIDs as well as percentage of fluoroquinolones (J01MA) of total antibiotic use.

A number of these indicators are due to concerns with the over use of co-amoxiclav, macrolides, third and fourth generation cephalosporins as well as fluoroquinolones (Adriaenssens et al., 2011a; Malo et al., 2014; WHO EUROPE, 2017).

Since multiple initiatives and reforms were instigated over time in the Republic of Srpska with varying intensity, coupled with the nature of reported figures, it was impossible to undertake sophisticated statistical analyses such as time series analyses. As a result, more simple statistical tests, including trend analyses, were performed to assess the level of significance, with significance seen as *p* < 0.05. This is similar to other health authority analyses where multiple interventions are conducted over time with no opportunity for time series analyses (Godman et al., 2010, 2014; Voncina et al., 2011; Bennie et al., 2012; Abilova et al., 2018).

No specific ethical approval was sought as only aggregated anonymized data was used for analysis, with Ministry of Health personnel involved in the study. This is similar to other studies of this nature (Godman et al., 2010, 2014; Bennie et al., 2012; Abilova et al., 2018).

## Results

**Figure 1:**
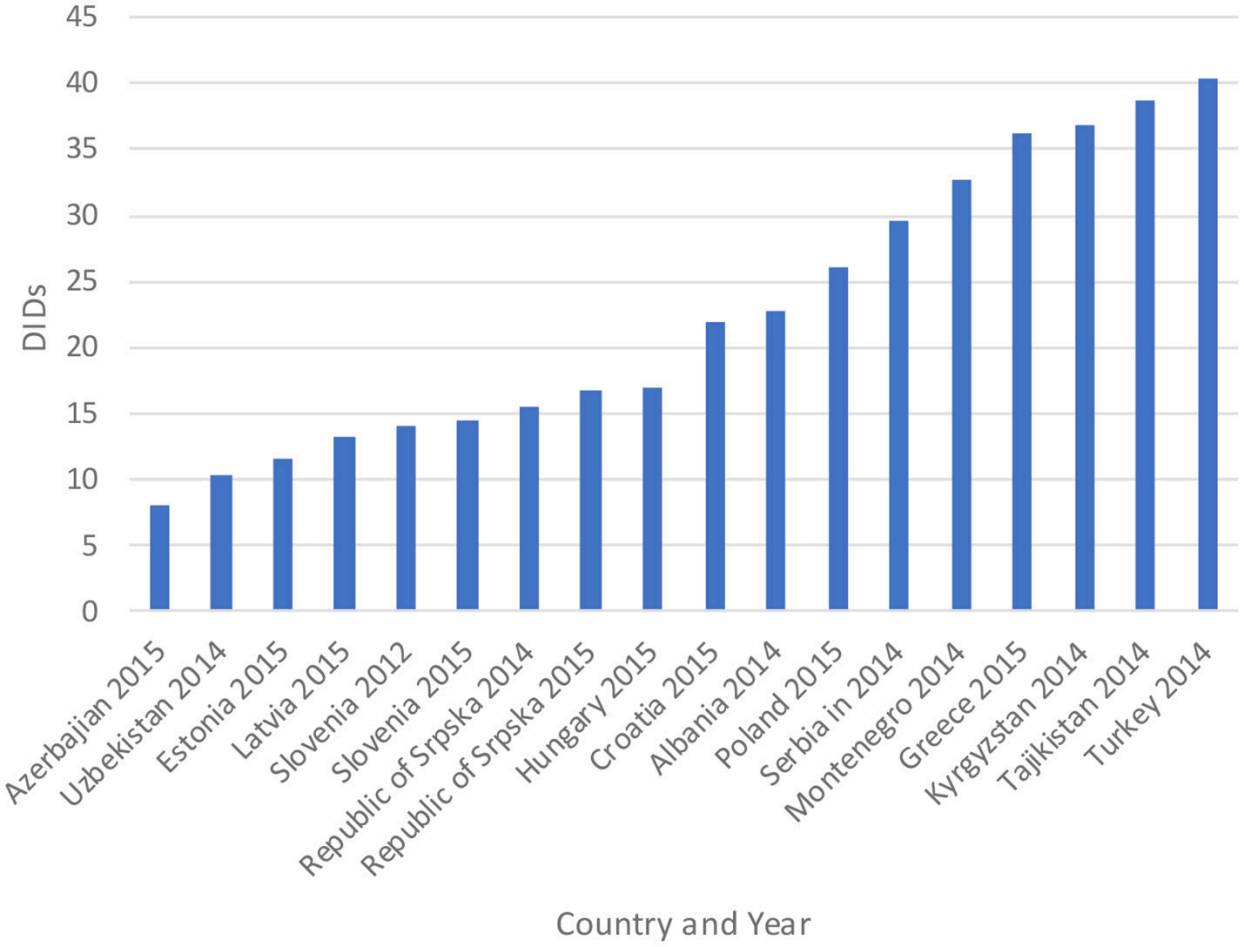
Antibiotic utilization in the Republic of Srpska in 2014 and 2015 (in DIDs) vs. neighboring countries in similar years (Taken from Fürst et al., 2015; WHO EUROPE, 2017; ECDC, 2018).

The total utilization of antibiotics for systemic use (J01 group) comprised 98% of total utilization of anti-infectives (J group). Utilization of the J01 group varied between 15.6 and 18.4 DIDs during the observed period (Tables 1, **4**), lower than a number of neighboring countries (Figure 1).

**Table 1:** Total utilization of antibiotics for systemic use (J01 group) expressed in DIDs and percentages from 2010 to 2015.

**ATC group**	**2010**		**2011**		**2012**		**2013**		**2014**		**2015**		***p*****-values**	
	**DIDs**	**%**	**DIDs**	**%**	**DIDs**	**%**	**DIDs**	**%**	**DIDs**	**%**	**DIDs**	**%**	**DID**	**%**
J01C	8.18	46.5	8.34	47.6	7.97	50.6	9.97	54.2	7.86	50.3	8.16	48.7	*P* = 0.16	*P* = 0.37
J01D	3.01	17.2	2.45	14.0	2.10	13.4	2.63	14.3	2.06	13.2	2.27	13.5	*P* = 0.18	*P* = 0.109
J01M	1.46	8.3	1.64	9.4	1.46	9.3	1.51	8.2	1.61	10.3	1.71	10.2	*P* = 0.35	*P* = 0.15
J01F	1.70	9.7	1.78	10.2	1.49	9.5	1.70	9.3	1.55	9.9	1.61	9.6	*P* = 0.39	*P* = 0.6
J01A	1.69	9.6	1.55	8.9	1.39	8.9	1.31	7.1	1.27	8.1	1.60	9.5	*P* = 0.12	*P* = 0.6
J01E	1.20	6.8	1.41	8.0	1.05	6.7	1.05	5.7	1.03	6.6	1.03	6.2	*P* = 0.74	*P* = 0.23
J01X	0.24	1.4	0.25	1.4	0.23	1.5	0.18	1.0	0.21	1.3	0.30	1.8	*P* = 0.44	*P* = 0.63
J01G	0.09	0.5	0.10	0.5	0.03	0.2	0.05	0.3	0.04	0.3	0.08	0.5	*P* = 0.51	*P* = 0.7
Total J01	17.6	100	17.5	100	15.7	100	18.4	100	15.6	100	16.8	100		

*J01A, Tetracyclines; J01C, Beta-lactam antibacterials, penicillins; J01D, Other beta-lactam antibacterials (cephalosporins); J01E, Sulfonamides and trimethoprim; J01F, Macrolides, lincosamides and streptogamins; J01G, Aminoglycoside antibacterials; J01M, Quinolone antibacterials; J01X, Other antibacterials; % = % of antibiotics in that class for the year*.

Figure 1 compares the utilization in recent years with other neighboring countries.

**Figure 2:**
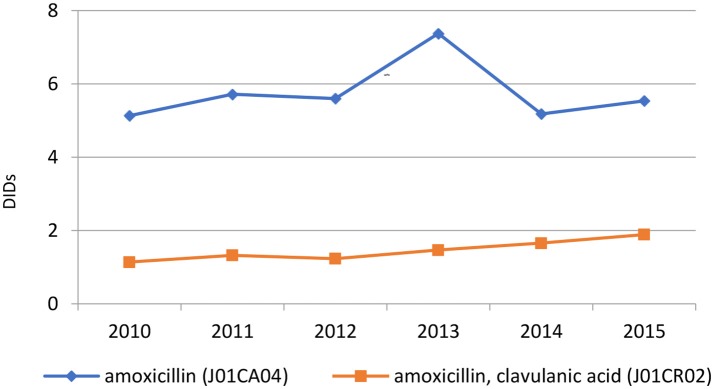
Amoxicillin vs. co-amoxiclav utilization expressed in DIDs from 2010 to 2015.

The penicillins (J01C) were the most consumed antibiotics comprising 50% of total antibiotic utilization on average, followed by other beta-lactam antibiotics (14%). The highest utilization of penicillins was observed in 2013 with 10.0 DIDs, while the lowest (7.9 DIDs) observed in 2014. The utilization of broad-spectrum penicillins (J01CA) was 6.04 DIDs on average (Table 2). Amoxicillin constituted 95% of this group (5.76 DIDs) (Figure 2), followed by ampicillin (0.28 DIDs). Co-amoxiclav (J01CR02) ranged from 1.1 to 1.9 DIDs from 2010 to 2015 (Figure 2), while beta-lactamase sensitive penicillins (J01CE) comprised 5% of total antibiotic use (Table 2), with phenoxymethylpenicillin the most prescribed antibiotic of this group.

**Table 2:** Utilization of penicillins (J01CA) expressed in DIDs from 2010 to 2015.

**ATC group**	**Pharmacological group**	**DIDs**						***P*-values**
		**2010**	**2011**	**2012**	**2013**	**2014**	**2015**	
J01CA	Penicillins with extended spectrum	5.62	6.10	5.88	7.56	5.38	5.72	*P* = 0.99
J01CR	Combinations of penicillins, incl. beta-lactamase inhibitors	1.14	1.33	1.23	1.47	1.66	1.89	*P* = 0.004
J01CE	Beta-lactamase sensitive penicillins	1.42	0.91	0.85	0.94	0.82	0.56	*P* = 0.03
J01CF	Beta-lactamase resistant penicillins	001	<0.01	<0.01	<0.01	<0.01	<0.01	*P* = 0.918
Total J01C		8.2	8.3	8.0	10.0	7.9	8.2	

Utilization of cephalosporins (J01D) decreased from 3.0 DIDs in 2010 to 2.3 DIDs in 2015 (Tables 1, **4**). The first-generation cephalosporins (J01DB) were the most used within J01D group (67.6%), of which cefalexin was the only one used. Its utilization decreased from 2.0 DIDs in 2010 to 1.5 DIDs in 2015, which represents a reduction of 25%. Overall, utilization of the second and third generation of cephalosporins (J01DC and J01DD) also declined during this period. Regarding the second-generation cephalosporins, cefuroxime had very stable utilization ranging from 0.45 to 0.56 DIDs during the study period, while utilization of cefaclor decreased from 0.15 in 2010 to 0.05 DIDs in 2015 (67%). Overall, third generation cephalosporins constituted 7.9% of total cephalosporins, or 1.2% on average of total antibiotics (Table **4**). After declining from 2010 to 2014, utilization of third generation cephalosporins subsequently increased in 2015 (Table **4**). The fourth generation cephalosporins, monobactams, and carbapenems, were not used at all.

Erythromycin was the most used macrolide (0.68 DIDs) followed by azithromycin (0.57 DIDs) and clarithromycin (0.32 DIDs). Overall, macrolides constituted 9.4% of total antibiotic utilization, which is about five times lower than the penicillins. The use of the macrolides was highest in 2011 (1.78 DIDs) and then dropped thereafter to 1.55 DIDs in 2014 (Table 1).

Doxycycline was the most used tetracycline (J01A), with quinolone utilization (J01M) ranging from 1.5 DDDs in 2010 to 1.7 DIDs in 2015 (Table 1), of which 90% were fluoroquinolones. Ciprofloxacin was the most prescribed fluoroquinolone. The only sulphonamide (J01E) was a combination of sulfamethoxazole with trimethoprim, which typically accounted for 7% of total antibiotic utilization (Table 1). Utilization of aminoglycoside antibiotics (J01G) and other antibiotics (J01X) was negligible (Table 1).

DU90% shows the number of antibiotics included in 90% of total utilization. Over the observed period, 10 antibiotics were constantly included in the DU90% profile. DU90% included 10 antibiotics in 2013, 11 in 2014, and 12 during the rest of the study years. Amoxicillin, amoxicillin with clavulanic acid (co-amoxiclav), cephalexin, ciprofloxacin, doxycycline, and sulfamethoxazole with trimethoprim were the most used antibiotics over the observed period (Table 3).

**Table 3:** Antibiotic utilization within Drug Utilization (DU90%) profile, expressed in DIDs from 2010 to 2015.

	**2010**		**2011**		**2012**		**2013**		**2014**		**2015**	
	**DID**	**%**	**DID**	**%**	**DID**	**%**	**DID**	**%**	**DID**	**%**	**DID**	**%**
Amoxicillin	5.14	29.26	5.72	32.67	5.60	35.59	7.38	40.12	5.19	33.20	5.54	33.07
Amoxicillin, clavulanic acid	1.14	6.49	1.33	7.60	1.23	7.82	1.47	7.99	1.66	10.62	1.89	11.28
Cefalexin	2.05	11.67	1.58	9.02	1.31	8.32	1.93	10.49	1.44	9.21	1.53	9.13
Ciprofloxacin	0.96	5.46	1.13	6.45	1.04	6.61	1.11	6.03	1.20	7.68	1.23	7.34
Doxycycline	1.61	9.16	1.48	8.45	1.22	7.75	1.20	6.52	1.11	7.10	1.20	7.16
Sulfamethoxazole, trimethoprim	1.20	6.83	1.41	8.05	1.05	6.67	1.05	5.71	1.03	6.59	1.03	6.15
Azithromycin	0.30	1.71	0.64	3.65	0.50	3.18	0.56	3.04	0.63	4.03	0.76	4.54
Phenoxymethyl-penicillin	1.17	6.66	0.71	4.05	0.66	4.19	0.74	4.02	0.55	3.52	0.53	3.16
Cefuroxime	0.49	2.79	0.56	3.20	0.51	3.24	0.54	2.94	0.48	3.07	0.45	2.69
Erythromycin	1.07	6.09	0.71	4.05	0.61	3.88	0.74	4.02	0.55	3.52	0.43	2.57
Clarithromycin	–	–	0.35	2.00	0.33	2.10	–	–	0.30	1.92	0.35	2.10
Ampicillin	0.48	2.73	0.38	2.17	0.28	1.78	–	–	–	–	–	–
Norfloxacin	0.30	1.71	–	–	–	–	–	–	–	–	–	–
Ambroxol, doxycycline	–	–	–	–	–	–	–	–	–	–	0.38	2.27
DU90	1–12		1–12		1–12		1–10		1–11		1–12	
%	90.56		91.36		91.13		90.88		90.46		91.46	
Others	13–32		13–31		13–31		11–30		12–30		13–30	
%	9.44		8.64		8.87		9.12		9.54		8.54	
Total	1–32		1–31		1–31		1–30		1–30		1–30	
%	100		100		100		100		100		100	

According to the quality indicators, the total utilization of beta-lactam antibiotics (penicillins and cephalosporins), macrolides and fluoroquinolones, expressed as percentage of total antibiotic use, was very stable over the observed period (Table 4). However, the use of co-amoxiclav increased from 6.5% of total antibiotic utilization in 2010 to 11.3% in 2015, which is a concern if this trend continues. At the same time, the use of erythromycin decreased for 37.2% within the macrolide group, while azithromycin rose proportionally (Tables 3, 4).

**Table 4:** Quality indicators for antibiotic utilization in the Republic of Srpska from 2010 to 2015.

**Quality indicator**	**2010**	**2011**	**2012**	**2013**	**2014**	**2015**
Total utilization of antibiotics (J01) (DIDs)	17.6	17.5	15.7	18.4	15.6	16.8
Total utilization of beta-lactam antibiotics (penicillins—J01C and cephalosporins—J01D) as a % of total antibiotic use	63.7	61.6	64.0	68.5	63.5	62.3
Total utilization of penicillins (J01C) as a % of total antibiotic use	46.5	47.6	50.6	54.2	50.3	48.7
Utilization of combination penicillins (co-amoxiclav—J01CR02) as % of total antibiotic use	6.5	7.6	7.8	8.0	10.6	11.3
Total utilization of cephalosporins—J01D (DIDs)	3.0	2.5	2.1	2.6	2.1	2.3
Utilization of 3rd and 4th generation cephalosporins (J01DD and J01DE) as % of total antibiotic use	1.9	1.1	1.3	0.6	0.6	1.4
Utilization of 3rd and 4th generation of cephalosporins as % of total cephalosporin use	11.0	7.7	9.5	4.1	4.6	10.4
Total utilization of macrolides, lincosamides and streptogramins- J01F (DIDs)	1.7	1.8	1.5	1.7	1.6	1.6
Total utilization of erythromycin (J01FA01), clarithromycin (J01FA09) and azithromycin (J01FA10) as % of total antibiotic use	9.3	9.7	9.1	8.9	9.4	9.2
Total utilization of erythromycin as % of total macrolide use, with corresponding increase in clarithromycin and azithromycin	64.7	40.9	42.0	45.0	36.7	27.5
Total utilization of quinolones J01M—(DIDs)	1.5	1.6	1.5	1.5	1.6	1.7
Total utilization of fluoroquinolones (J01MA) as % of total antibiotic utilization	7.4	8.4	8.4	7.4	9.3	9.4

**Figure 3:**
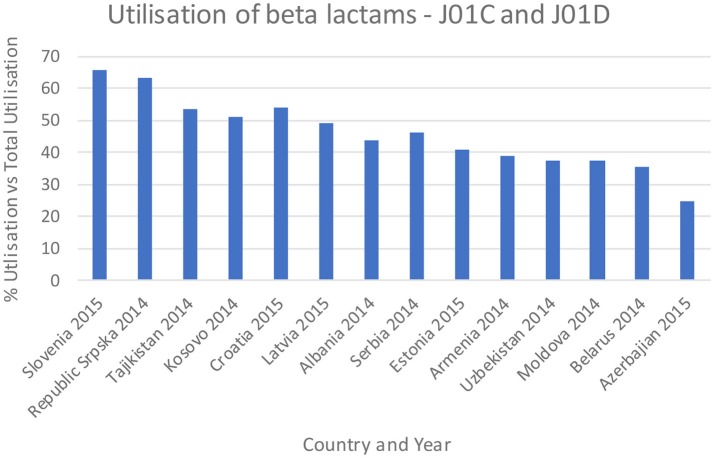
Percentage utilization of beta-lactams as a % of total antibiotic utilization across countries (taken from Fürst et al., 2015; WHO EUROPE, 2017; ECDC, 2018).

**Figure 4:**
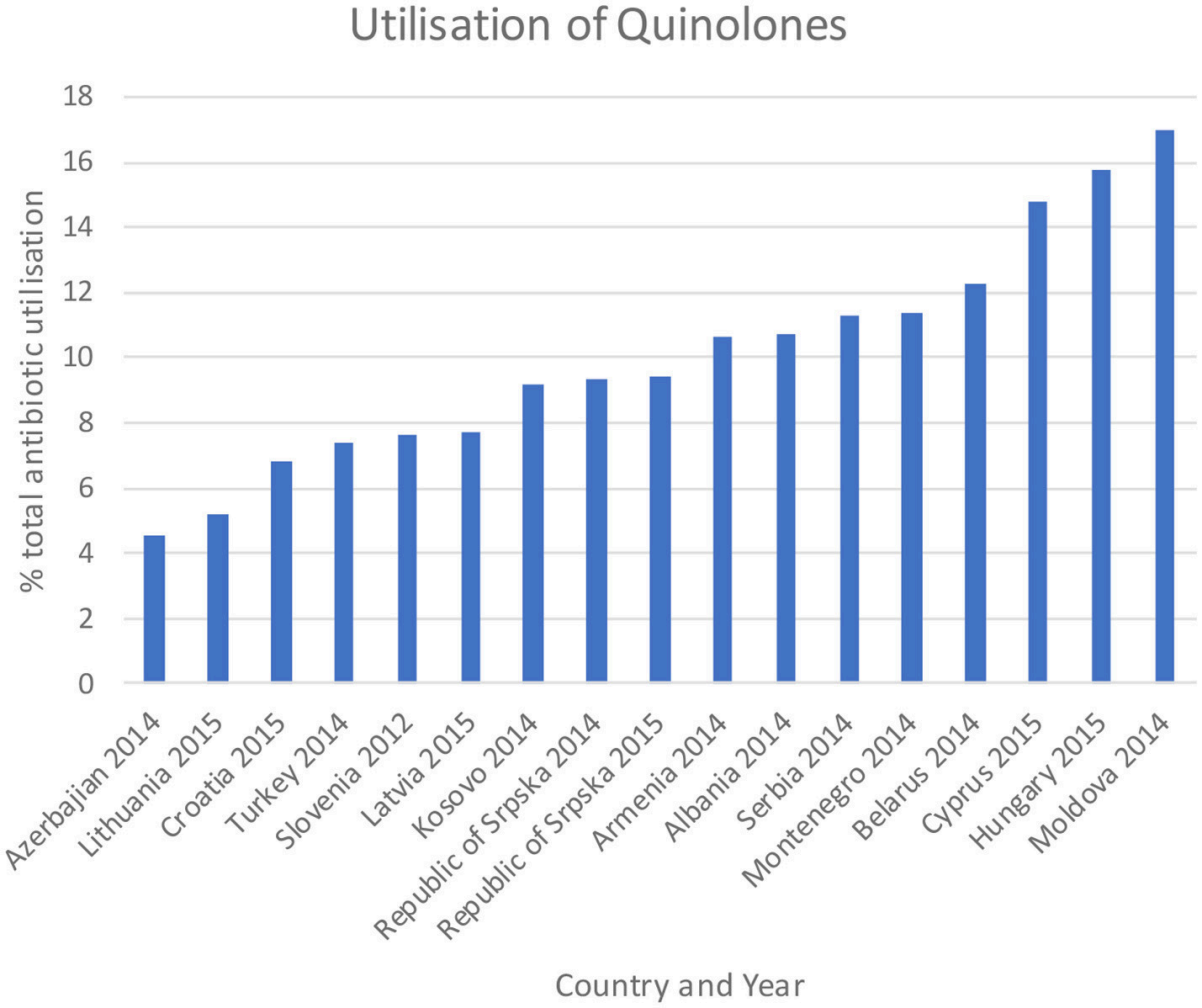
Utilization of quinolones as a % of total antibiotic utilization across countries (taken from Fürst et al., 2015; WHO EUROPE, 2017; ECDC, 2018).

**Figure 5:**
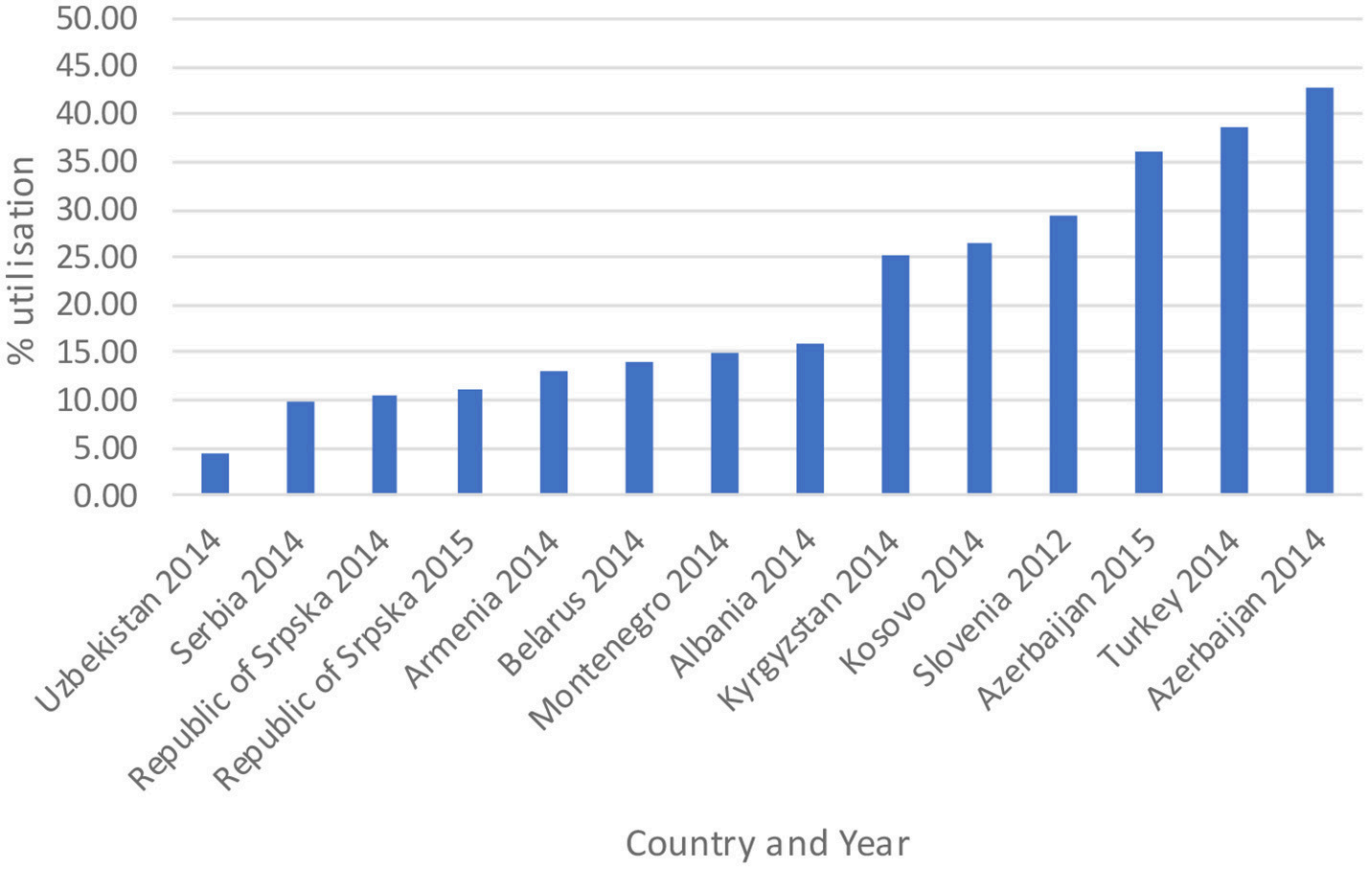
Percentage combination penicillins (co-amoxiclav J01CR02) as a % of total antibiotic utilization (taken from Fürst et al., 2015; WHO EUROPE, 2017; ECDC, 2018).

Figures 3–**5** and Table 5 provide comparisons with similar and neighboring countries to the Republic of Srpska in recent years.

**Table 5:** Utilization of third and fourth generation cephalosporins as a % of total cephalosporins (J01D) (taken from Fürst et al., 2015; WHO EUROPE, 2017; ECDC, 2018).

**Country and year**	**%**
Republic of Srpska 2014	4.6
Republic of Srpska 2015	10.4
Serbia 2014	16
Albania 2014	30
Turkey 2014	38
Montenegro 2014	40
Moldova 2014	46
Uzbekistan 2014	63
Azerbaijan 2014	76
Belarus 2014	79
Kyrgyzstan 2014	81
Azerbaijan 2015	82

## Discussion

Total antibiotic utilization was relatively constant during the study period in the Republic of Srpska (Table 1), with minor fluctuations, and an overall a decline in total utilization of 5% between 2010 and 2015, with a corresponding reduction in expenditure on antibiotics (J01) in 2013 vs. 2009 (Petrusic and Jakovljevic, 2015). In addition, antibiotic utilization in the Republic was lower than a number of neighboring countries and former Soviet Union Republics in recent years (Figure 1). This is in direct contrast with total worldwide antibiotic utilization, which rose by 36% during the past decade (Laxminarayan et al., 2016). There appears to be several reasons for this in the Republic of Srpska. Firstly, health care in the Republic is well-regulated by a number of policies, strategies, guidelines, and normatives (Boxes 1,2), despite limited available resources for such initiatives. The Law on health care and the Law of health insurance enables all patients to have adequate health care access in the Republic, including medicines and other types of treatment (MoHSW, 1999, 2009). Standard treatment guidelines for the most common clinical problems in ambulatory care were developed by family physician associations and adopted by Ministry of Health and Social Welfare (MoHSW) in 2004 in order to improve the quality and rationality of physician prescribing. Based on these guidelines, only recommended and essential antibiotics were included in the HIF positive list of medicines to emphasize rational prescribing and reduce unnecessary expenditure to help ensure cost-efficient antibiotics are available for all citizens in the Republic. This approach is similar to the situation seen in some European countries and regions with multiple initiatives to enhance the quality and efficiency of prescribing (Gustafsson et al., 2011; Bjorkhem-Bergman et al., 2013). However, we are aware that there is variable implementation of guidelines among a number of European countries in practice (Francke et al., 2008; Sermet et al., 2010; van Dijk et al., 2011; Brusamento et al., 2012; Fitzgerald et al., 2014; Baker et al., 2015). In addition, as mentioned, there are concerns with issues of evidence based medicine among physicians in the Balkan countries (Jakovljevic et al., 2016a,b); however, this appears not to be the case in the Republic of Srpska certainly with respect to antibiotic prescribing.

Secondly, a number of initiatives and activities have been implemented in the Republic in order to improve knowledge, attitudes and practices of pharmacists to reduce antibiotic self-medication and improve appropriate management of patients with URTIs (Boxes 1, 2), especially as pharmacists are often the first health professional patients see with their URTI (Marković-Peković et al., 2017). This includes the tightening of self-medication regulations, with self-medication known to appreciably increase antibiotic utilization in URTIs and similar conditions (Kalaba et al., 2011; Kibuule et al., 2016; Marković-Peković et al., 2017). This is different to Albania where self-medication is common particularly for URTIs, and there are considerable concerns with pharmacy knowledge regarding antibiotics and viruses (Jorgji et al., 2014; Hoxha et al., 2015, 2018). There are also concerns with continuing self-medication in Serbia (Kalaba et al., 2011). Education among pharmacists also reduced the extent of self-medication with antibiotics in Kenya especially for URTIs (Mukokinya et al., 2018).

These combined initiatives also appeared to result in high use of beta-lactam antibiotics and limited use of third and fourth generation cephalosporins and fluorquinolones in the Republic in recent years in accordance with agreed quality indicators (Table 4), comparable or improved prescribing vs. neighboring countries (Figures 3, 4, and Table 5). We have also seen reduced expenditure on first and second generation cephalosporins in Serbia between 2007 and 2012, although also reduced prescribing of combinations of penicillins (J01CR) and penicillins with extended spectrums (J01CA) (Jakovljevic and Souliotis, 2016). The DU90% profile (Table 3) shows that first and second generation antibiotics were mainly used, reflecting the antibiotics reimbursed by HIF and contained in current clinical guidelines (Box 2). This is similar to the situation with other medicines when prescribing is restricted and/or where there are high co-payments to rationalize pharmacotherapy (Sakshaug et al., 2007; Martikainen et al., 2010; Wettermark et al., 2010; Kalaba et al., 2012; Markovic-Pekovic et al., 2012; Moon et al., 2014). In Slovenia, prescribing restrictions for co-amoxiclav, the cephalosporins, macrolides, and fluoroquinolones, also significantly reduced their use (Fürst et al., 2015). These findings support other published studies which suggest that multiple initiatives are typically needed to positively influence prescribing (Bero et al., 1998; Barton, 2001; Francke et al., 2008; Godman et al., 2013, 2014; Moon et al., 2014).

Amoxicillin, which is included in the HIF positive A list, was the most utilized penicillin in the Republic of Srpska, with utilization of co-amoxiclav approximately 4 times lower than amoxicillin. The low utilization of co-amoxiclav in the Republic compares favorably with neighboring countries (Figure 5). However, there are concerns with the rise in its utilization in recent years (Figure 2 and Tables 2, 4). This is due to concerns with increasing side-effects and resistance leading to co-amoxiclav now being recommended as a second line antibiotic after amoxicillin in a number of guidelines (Andrade and Tulkens, 2011; Desrosiers et al., 2011; NHS Scotland, 2014; GCG prescribing, 2017; ECDC, 2018). There are also concerns with currently limited prescribing of phenoxymethyl penicillin in the Republic (Table 3) as it is likely that a high proportion of infections seen in ambulatory care are likely to be URTIs (Llor and Bjerrum, 2014; Rezal et al., 2015; Dyar et al., 2016). These will be areas to address if these trends (Figure 2) continue.

Encouragingly, given concerns with the development and spread of resistant strains of *C. difficle* (Dancer, 2001; Bätzing-Feigenbaum et al., 2016), there were low rates of cephalosporin utilization, with rates remaining relatively constant over the study period (Tables 1, 4). In addition, as mentioned, the Republic of Srpska had low utilization of third and fourth generation of cephalosporins in recent years (Table 4), comparing favorably with neighboring countries (Table 5). There was also relatively low utilization of macrolides (J01F), with rates decreasing in recent years but not reaching statistical significance (Tables 1, 4). This is similar to Serbia with expenditure on macrolides falling in recent years (Jakovljevic and Souliotis, 2016). However, the prescribing of azithromycin is increasing, with a simultaneous decrease in utilization of erythromycin (Tables 3, 4), resulting in the utilization of azithromycin exceeding erythromycin in 2014 and 2015. This is causing concern with increasing resistance rates as well as side-effects with the macrolides (Albert and Schuller, 2014; ECDC, 2018).

Concerns with the recent increased utilization of co-amoxiclav and azithromycin, as well as higher use of fluoroquinolones than some neighboring countries (Table 1), has already resulted in planned activities, with their implementation and impact being monitored. These ongoing and planned activities (Box 2) should further help with enhancing appropriate antibiotic use in the Republic of Srpska. Planned activities also include programmes to improve inter-sectoral control over antibiotic utilization, including both health and veterinary sectors, as well as improved undergraduate and postgraduate education regarding antibiotics and AMR. The development of specific indicators for monitoring antibiotic utilization is also planned, building on existing quality indicators for NCDs, as well as monitoring hospital resistance patterns. These data will be used to revise and update the clinical guidelines for rational antibiotic use in the Republic. Planned activities also include improving the information technology for monitoring of AMR and antibiotic consumption. The findings will subsequently be shown to health professionals to emphasize what has been done so far and to discuss further activities. This is similar to activities in for instance in Sweden with its monitoring of prescribing in the regions together with the development of quality indicators to improve physician prescribing practices (Godman et al., 2009; Wettermark et al., 2009; Gustafsson et al., 2011; Eriksen et al., 2017).

Alongside this, pharmacists and family physicians will be encouraged to communicate more to their patients about rational antibiotic use and AMR, building on current initiatives. This will be combined with planned activities to further raise public awareness regarding the potential harmful effects of excessive, inappropriate, and unnecessary use of antibiotics. These programmes will also be the subject of future research projects.

We believe this study clearly shows that even lower income countries such as the Republic of Srpska can appreciably improve rational medicine utilization by introducing multiple interventions and initiatives, acting as an exemplar to other European countries and wider.

We are aware of a number of limitations of this study. These include no access to prescribing data to be able to assess the quality of antibiotic prescribing against standard treatment guidelines. However, we believe our methodology, including the use of total antibiotic utilization via the PHI datasets, as well as comparisons with neighboring countries, adds robustness to our findings and conclusions.

In conclusion, without looking specifically at prescribing indications, we believe there appears to be rational antibiotic utilization in the Republic of Srpska in recent years compared to neighboring countries, with favorable use of penicillins combined with moderate or low use of co-amoxiclav, cephalosporins including third and fourth generation cephalosporins, macrolides, and quinolones. This again demonstrates that multiple interventions with key stakeholder groups can favorably improve medicine utilization patterns.

## Author contributions

LB, VM-P, MÐ, RŠ, JB, and BG: developed the concept; LB, AK, VM-P, RŠ, and BG: undertook the analysis and the first write-up of the manuscript. All authors critiqued the initial and subsequent drafts before submission.

### Conflict of interest statement

VM-P is employed by the Ministry of Health and Social Welfare in the Republic of Srpska and LB, MÐ, and JB are employed by the Public Health Institute. The other authors declare that the research was conducted in the absence of any commercial or financial relationships that could be construed as a potential conflict of interest.
